# Supplementary material for: The Gut Microbiome Alterations in Pediatric Patients with Functional Abdominal Pain Disorders
Source: Microorganisms. 2021 Nov 15;9(11):2354. doi: 10.3390/microorganisms9112354 (PMC8622193; doi:10.3390/microorganisms9112354)
Supplement: Supplementary file 1 [file microorganisms-09-02354-s001.zip › Supplementary File S1.pdf]

Questionnaire on Pediatric Gastrointestinal Symptoms, Rome IV Version (QPGS-RIV)

(Adapted from the Questionnaire on Pediatric Gastrointestinal Symptoms, Walker, Caplan-Dover, & Rasquin-Weber, 2000)

Self-Report Form for Children and Adolescents (10 years of age and older)

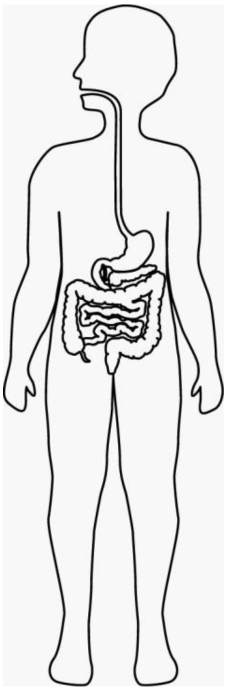

Instructions

This questionnaire is about your digestive system (esophagus, stomach, small intestine, and colon) and problems you can have with it. Certain problems may apply to you and others will not.

Please try to answer all of the questions as best as you can.

If you have any questions, the research assistant will be glad to help!

Section A. Pain and Uncomfortable Feelings Above the Belly Button

The dotted line in the picture below shows an area ABOVE your belly button where children sometimes hurt, feel pain, or have an uncomfortable feeling. Some words for these feelings are stomachaches, nausea, bloating, or feeling full after eating very little.

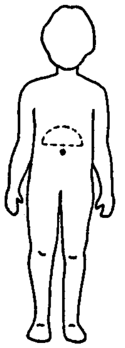

Above the Belly Button

The questions in this section are about pain and uncomfortable feelings ABOVE the belly button that you may have had in the past month. Children can have pain and uncomfortable feelings in more than one area of the belly. In different parts of the questionnaire, you will be asked about other areas in your belly.

1. In the past month, on how many days did you have pain, stomach ache or belly ache *above the belly button* (even if it only lasted a small part of the day)?
   0.____ Never
   1.____ 1 day
   2.____ 2 days
   3.____ 3 days
   4.____ 4 days
   5.____ 5 days or more

2. In the past month, on how many days did you have burning in your chest (even if it only lasted a small part of the day)?
   0.____ Never
   1.____ 1 day
   2.____ 2 days
   3.____ 3 days
   4.____ 4 days
   5.____ 5 days or more

3. In the past month, on how many days did you feel uncomfortably full or have nausea or bloating after a normal sized meal (the amount you usually eat)?
   0.____Never
   1.____1 day
   2.____2 days
   3.____3 days
   4.____4 days
   5.____ 5 days or more

4. In the past month, on how many days were you NOT able to finish your meal because you felt too full?
   0.____Never
   1.____1 day
   2.____2 days
   3.____3 days
   4.____4 days
   5.____ 5 days or more

If you have NOT had ANY pain or uncomfortable feelings in the past month (you answered Never to ALL questions 1-4), please go to Section B.

5. When you feel uncomfortable above your belly button (pain, stomach ache, belly ache, burning, feeling full etc.), do you also have any of the following feelings:
   (You may check one or more than one.)

a.	Bloating (your belly sticking out more than usual)	0.____No	1._____Yes
b.	Nausea (feeling sick to your stomach or like you may throw up)	0.____No	1._____Yes
c.	Burping or belching	0.____No	1._____Yes

6. When you feel uncomfortable above your belly button:

a. Does it get worse when you eat?	0.____No	1._____Yes
b. Does it get better when you eat?	0.____No	1._____Yes

7. How long have you felt uncomfortable above your belly button (pain, stomach ache, belly ache, burning, feeling full etc.)?
   1.____ Less than a month
   2.____ 2 months
   3.____ 3 months
   4. ____ 4-11 months
   5. ____ 1 year or longer

Circle a number for your answer to each question below.

**In the past month, when you hurt or felt uncomfortable above the belly button, how often:**	Never	Once in a while	Sometimes	Most of the time	Always
8. Did the pain or uncomfortable feeling happen around the time you pooped?	0	1	2	3	4
9. Were your poops softer and more mushy or watery than usual?	0	1	2	3	4
10. Were your poops harder or lumpier than usual?	0	1	2	3	4
11. Did you poop more often than usual?	0	1	2	3	4
12. Did you poop less often than usual?	0	1	2	3	4

Section B. Belly Aches and Abdominal Pain Around and Below the Belly Button

The questions in this section are about the areas AROUND and BELOW your belly button. These areas are shown with dotted lines in the pictures below. Children sometimes have a belly ache or pain in these areas. Belly aches are sometimes milder than pain. Some children call their belly aches or pains "stomach aches" or "tummy aches."

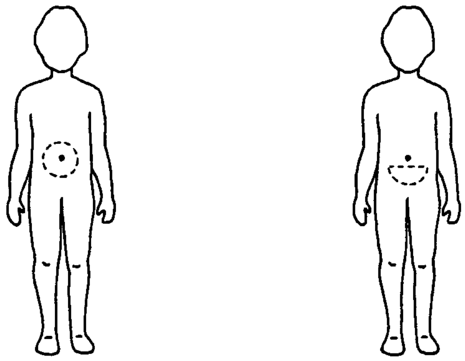

Around the Belly Button **Below the Belly Button**

1. In the past month, on how many days did you have a belly ache, stomach ache or pain *in the area around or below the belly button* (even if it only lasted a small part of the day)?
   0.____ Never
   1.____ 1 day
   2.____ 2 days
   3.____ 3 days
   4.____ 4 days
   5.____ 5 days or more

If you HAVE NOT had ANY belly aches or pain in the areas around or below the belly button in the past month, please go to Section C.

2. Do you feel the belly ache or pain around or below your belly button only:
   a. When eating or right after eating 0.___ No 1.___ Yes
   b. (For girls only): When you are having your period
   0.___ No 1.___ Yes 2.___ I have not started menstruation

3. How long have you had a belly ache, stomach ache or pain around or below your belly button?
   1.____ Less than a month
   2.____ 2 months
   3.____ 3 months
   4. ___ 4-11 months
   5. ___ 1 year or longer

Circle a number for your answer to each question below.					
**In the past month, when you had a belly ache, stomach ache or pain around or below the belly button, how often:**	Never	Once in a while	Sometimes	Most of the time	Always
4. Did the belly ache, stomach ache or pain happen around the time you pooped?	0	1	2	3	4
5. Were your poops softer and more mushy or watery than usual?	0	1	2	3	4
6. Were your poops harder or lumpier than usual?	0	1	2	3	4
7. Did you poop more often than usual?	0	1	2	3	4
8. Did you have poop less often than usual?	0	1	2	3	4

9. Are you taking medications for constipation or hard stools?
   1. ___ No
   2. ___ Yes
   3. ___ Don't know

   9a. If you answered Yes, how often does your belly ache, stomach ache or pain get better if you use these medications?
      a. ____ Never
      b. ____ Once in a while
      c. ____ Sometimes
      d. ____ Most of the time
      e. ____ Always

10. Do you ever get **severe intense pain** around the belly button that hurts so much it makes you **stop everything** that you are doing?
   0.____ No (*if no, please go to section C)*
   1.____ Yes

   10a. When the pain hurts so much that you stop everything you are doing, how long does it usually last?
      1. ____ Less than an hour
      2. ____ 1-2 hours
      3. ____ 3-4 hours
      4. ____ Most of the day
      5. ____ The whole day

10b. IN THE PAST 6 MONTHS how often did it hurt so much around your belly button that you stopped everything you were doing?

1._____ 1 time
2._____ 2 times
3._____ 3-5 times
4._____ 6 or more times

10c. When it hurt so much around your belly button that you stopped everything you were doing, did you have any of the following?

1. Not wanting to eat	0.______No	1.______Yes
2. Nausea (feeling sick to your stomach)	0.______No	1.______Yes
3. Vomiting (throwing up)	0.______No	1.______Yes
4. Pale skin	0.______No	1.______Yes
5. Headache	0.______No	1.______Yes
6. Eyes sensitive to light	0.______No	1.______Yes

10d. When the pain around your belly button stops, how long does it usually take for another episode of severe intense pain to start?

0. ____ several minutes
1. ____ several hours
2. ____ several days
3. ____ several weeks
4. ____ several months

Section C. Bowel Movements (“Poop,” “Stool,” “Number 2”)

This section asks about your bowel movements. There are many words for bowel movements, such as “poop,” “stool,” “BM’s,” and “going to the bathroom for number 2.” Your family may use another special word when they talk about pooping.

1. In the last month, how often did you usually poop?
   1._____ 2 times a week or less often
   2._____ 3-6 times a week
   3._____ Once a day
   4._____ 2-3 times a day
   5._____ More than 3 times a day

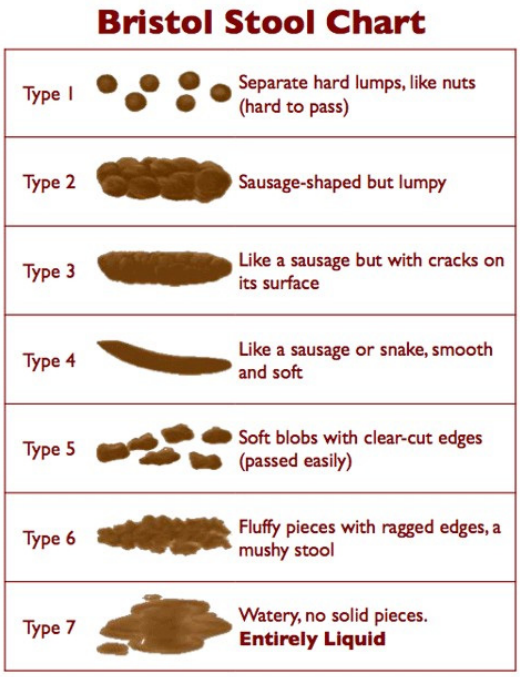

2. Look at the Bristol Stool Chart above. In the last month, what was your poop usually like?
   1.____Type 1
   2.____Type 2
   3.____Type 3
   4.____Type 4
   5.____Type 5
   6.____Type 6
   7.____Type 7
   8.____It depends (my poops are not always the same)

2a. If your poops were usually hard (type 1 or 2 on the pictures above), for how long have they been hard?
   0.____Less than 1 month
   1.____1 month
   2.____2 or more months
   3.____My poops are not usually hard

3. In the last month, did it hurt when you had a poop?

   0.____No
   1.____Yes

4. In the last month, did you have a poop that was so big that it clogged the toilet?
   0.____No
   1.____Yes

5. Some children hold in their poop even when there is a toilet they could use. They may do this by stiffening their bodies or crossing their legs. In the last month, while at home, how often did you try to hold in a poop?
   0.____ Never
   1.____ 1-3 times a month
   2.____ Once a week
   3.____ Several times a week
   4.____ Every day

6. Did a doctor or nurse ever examine you and say that you had a huge poop inside?
   0.____ No
   1.____ Yes

7. In the last month, how often did you poop in your pants?
   0.____ Never. *(If never, please go to Section D)*
   1.____ Less than once a month
   2.____ 1-3 times a month
   3.____ Once a week
   4.____ Several times a week
   5.____ Every day

   7a. When you pooped in your pants, how much did it stain or soil your underwear?
      1.______ Underwear was only stained (no poop)
      2.______ Small amount of poop in underwear (less than a whole poop)
      3.______ Large amount of poop in underwear (a whole poop)

   7b. For how long have you pooped in your pants?
      1.____ Less than a month
      2.____ 1 month
      3.____ 2 months
      4.____ 3-11 months
      5.____ 1 year or longer

Section D. Nausea and Vomiting

This section asks about feeling nauseated or vomiting. Nausea is when you feel sick to your stomach or feel like you may throw up. There are many words for nausea, such as "upset stomach," "feeling queasy," or "feeling sick to your stomach". Your family may use a special word when talking about nausea.

1. In the past month, how often did you feel nausea or sick to your stomach?
   0.____ Never *(If never, go to question 5)*
   1.____ 1-3 times a month
   2.____ Once a week
   3.____ Twice a week
   4.____ Three times a week or more often
   5. ____ Every day

2. How long have you felt nausea or sick to your stomach?
   1.____ Less than a month
   2.____ 2 months
   3.____ 3 months
   4. ___ 4-11 months
   5. ___ 1 year or longer

3. Does the nausea usually start after you eat? 0.______No 1.______Yes

4. When you have nausea, do you:
   1. Vomit 0.______No 1.______Yes
   2. Feel pain 0.______No 1.______Yes

   4a. If you have both pain and nausea, which one bothers you the most?
   (*Skip this question if you do not have both pain and nausea.)*
   1.______ Nausea
   2.______ Pain

The next questions are about vomiting. Vomiting is when food comes back from your stomach and out of your mouth. There are many words for vomiting such as "retching," "throwing up," "gagging" and "heaving." Your family may use another special word when they talk about vomiting.

5. In the past month, how often did you vomit or throw up?
   0.____ Never *(If never, go to question 7)*
   1.____ 1-3 times a month
   2.____ Once a week
   3.____ Several times a week
   4.____ Every day

6. How long have you had problems with vomiting or throwing up?
   1.____ Less than a month
   2.____ 2 months
   3.____ 3 months
   4. ____4-11 months
   5. ___ 1 year or longer

7. IN THE PAST 6 MONTHS, how many times did you vomit or throw up *again and again without stopping for two hours or longer***?**
   0.____ Never *(If never, go to question 8)*
   1.____ Once
   2.____ 2 times
   3.____ 3 times
   4.____ 4 or more times

   7a. How long have you had problems with vomiting or throwing up again and again without stopping?
      1._____ 1 month or less
      2._____ 2-3 months
      3._____ 4-5 months
      4._____ 6 months or longer

   7b. Do you usually feel nausea or sick to your stomach when you vomit or throw up again and again without stopping?
      0._____ No
      1._____ Yes

   7c. When the vomiting or throwing up stops, how long does it usually take for it to happen again?
      0._____ several minutes
      1._____ several days
      2._____ several weeks
      3._____ several months or longer

8. In the past month, how often did food come back up into your mouth after eating?
   0.____ Never *(If never, go to question 9)*
   1.____ 1-3 times a month
   2.____ Once a week
   3.____ Several times a week
   4.____ Every day

   8a. If food comes back into your mouth after eating, does this usually happen less than an hour after you eat?
      0._____ No
      1._____ Yes

   8b. Does it happen while you are sleeping?
      0._____ No
      1._____ Yes

   8c. Do you usually feel nausea, sick to your stomach or vomit and throw up when this happens?
      0._____ No
      1._____ Yes

   8d. What do you usually do with the food that comes back up into your mouth?
      0._____ Swallow it
      1._____ Spit it out

9. Do you ever make yourself vomit or throw up (for example, by sticking a finger in your throat)?

0._____ Never
1._____ Once in a while
2._____ Sometimes
3._____ Most of the time
4._____ Always

Section E. Other Symptoms

Circle a number for your answer to each question					
**In the past month, how often did you:**	Never	1-3 times a month	Once a week	Several times a week	Every day
1. Burp (belch) *again and again* without wanting to?	0	1	2	3	4
2. Pass a lot of gas (fart) *very frequently*?	0	1	2	3	4
3. Develop a swollen belly during the day (a belly that definitely sticks out more than usual)?	0	1	2	3	4
4. Swallow or gulp extra air? (You might hear a clicking noise when you swallow)	0	1	2	3	4
