# Supplementary material for: The Gut Microbiome Alterations in Pediatric Patients with Functional Abdominal Pain Disorders
Source: Microorganisms. 2021 Nov 15;9(11):2354. doi: 10.3390/microorganisms9112354 (PMC8622193; doi:10.3390/microorganisms9112354)
Supplement: Supplementary file 1 [file microorganisms-09-02354-s001.zip › Supplementary File S10.pdf]

Sample ID	T-score
1-001	70.8
1-002	57.1
1-003	60.1
1-004	51.6
1-005	59.1
1-006	37
1-007	42.6
1-008	64.9
1-009	54
1-011	63
1-012	63.9
1-015	37
1-016	NA
1-017	50.3
1-021	62
1-024	42.6
1-025	66.8
1-027	59.1
1-029	37
1-030	52.8
1-033	47.2
1-034	60.1
1-036	51.6
1-039	56.1
1-043	50.3
1-044	63.9
1-046	71.9
1-055	64.9
1-001c	77.2
1-002c	65.9
1-003c	69.8
1-005c	65.9
1-006c	NA
1-007c	57.1
1-011c	64.9
1-015c	45.2
1-016c	51.9
1-017c	43.5
1-021c	60.1
1-024c	55

1-025c	61
1-029c	37
1-030c	56.1
1-033c	56.1
1-039c	59.1
1-043c	50.3
1-044c	67.8
1-001d	75.6
1-002d	62
1-003d	NA
1-005d	65.9
1-006d	NA
1-007d	56.1
1-008d	63.9
1-011d	57.1
1-015d	37
1-016d	57.2
1-017d	37.6
1-021d	58.1
1-024d	63
1-025d	71.9
1-029d	37
1-030d	51.6
1-033d	47.2
1-036c	55
1-039d	60.1
1-043d	50.3
1-044d	66.8
1-046d	NA
1-055c	63.9
