# Supplementary material for: The Gut Microbiome Alterations in Pediatric Patients with Functional Abdominal Pain Disorders
Source: Microorganisms. 2021 Nov 15;9(11):2354. doi: 10.3390/microorganisms9112354 (PMC8622193; doi:10.3390/microorganisms9112354)
Supplement: Supplementary file 1 [file microorganisms-09-02354-s001.zip › Supplementary File S2.pdf]

Questionnaire on Pediatric Gastrointestinal Symptoms, Rome IV Version (QPGS-RIV)

(Adapted from the Questionnaire on Pediatric Gastrointestinal Symptoms, Walker, Caplan-Dover, & Rasquin-Weber, 2000)

Parent-Report Form for Children and Adolescents (4 years of age and older)

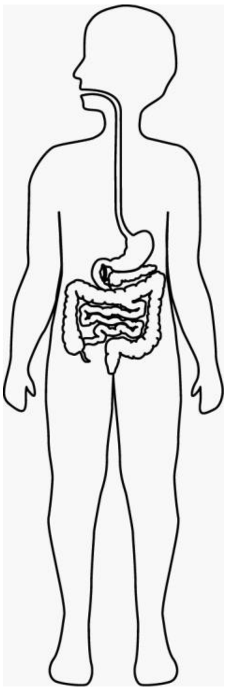

Instructions

This questionnaire is about your child's digestive system (esophagus, stomach, small intestine, and colon) and problems they can have with it. Certain problems may apply to your child and others may not.

Please try to answer all of the questions as best as you can.

Please let us know if you have any questions, we'll be happy to help!

Section A. Belly ache and Uncomfortable Feelings Above the Belly Button

The dotted line in the picture below shows an area ABOVE your child's belly button where children sometimes hurt, feel pain, or have an uncomfortable feeling. Some words for these feelings are stomach aches, nausea, bloating, or feeling full after eating very little.

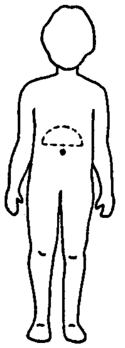

Above the Belly Button

The questions in this section are about pain and uncomfortable feelings ABOVE the belly button that your child may have had in the past month. Children can have pain and uncomfortable feelings in more than one area of the belly. In a different section of the questionnaire, you will be asked about the areas around and below your child's belly button.

1. In the past month, on how many days did your child have pain, stomach ache or belly ache in the upper abdomen *above the belly button* (even if it only lasted a small part of the day)?
   0._____ Never
   1.____ 1 day
   2.____ 2 days
   3.____ 3 days
   4.____ 4 days
   5.____ 5 days or more

2. In the past month, on how many days did your child have heartburn or a burning sensation in their chest (even if it only lasted a small part of the day)?
   0.____ Never
   1.____ 1 day
   2.____ 2 days
   3.____ 3 days
   4.____ 4 days
   5.____ 5 days or more

3. In the past month, on how many days did your child feel uncomfortably full, or feel nauseated or bloated after a normal sized meal (the amount your child usually eats)?
   0.____ Never
   1.____ 1 day
   2.____ 2 days
   3.____ 3 days
   4.____ 4 days
   5.____ 5 days or more

4. In the past month, on how many days was your child NOT able to finish a normal sized meal because he/she felt too full?
   0.____ Never
   1.____ 1 day
   2.____ 2 days
   3.____ 3 days
   4.____4 days
   5.____ 5 days or more

If your child has not had ANY pain or uncomfortable feelings in the past month (answered Never to ALL questions 1-4) please go to Section B.

5. When your child feels uncomfortable *above his or her belly button (*pain, stomach ache, belly ache, burning, feeling full, etc.), does he/she also have any of the following feelings: (you may check one or more than one).

a. Bloating (your child's belly is unusually large) 0._____ No 1.______ Yes
b. Nausea (feeling sick to his/her stomach) 0._____ No 1.______ Yes
c. Burping or belching 0._____ No 1.______ Yes

6. When your child feels uncomfortable above his or her belly button:
a. Does it get worse with eating? 0._____ No 1.______ Yes
b. Does it get better with eating? 0._____ No 1.______ Yes

7. How long has your child had pain or other uncomfortable feelings (heartburn, feeling full, etc.) above the belly button?
   1.____less than a month
   2.____2 months
   3.____3 months
   4. ___4-11 months
   5. ___1 year or longer

Circle a number for your answer to each question below.

**In the past month, when your child hurt or felt uncomfortable above the belly button, how often:**	Never	Once in a while	Some-times	Most of the time	Always
8. Did the pain or uncomfortable feeling happen around the time your child pooped?	0	1	2	3	4
9. Were your child's poops softer and more mushy or watery than usual?	0	1	2	3	4
10. Were your child's poops harder or lumpier than usual?	0	1	2	3	4
11. Did your child poop more often than usual?	0	1	2	3	4
12. Did your child poop less often than usual?	0	1	2	3	4

Section B. Belly Aches and Abdominal Pain Around and Below the Belly Button

The questions in this section are about the areas AROUND and BELOW your child's belly button. These areas are shown with dotted lines in the pictures below. Children sometimes have a belly ache or pain in these areas. Belly aches are sometimes milder than pain. Some children call their belly aches or pains "stomach aches" or "tummy aches."

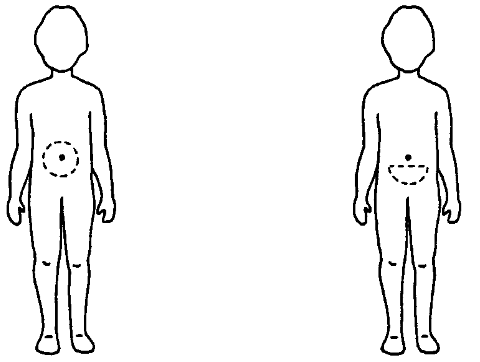

Around the Belly Button **Below the Belly Button**

1. In the past month, on how many days did your child have a belly ache, stomach ache or pain *in the area around or below the belly button* (even if it only lasted a small part of the day)?
   0.____ Never
   1.____ 1 day
   2.____ 2 days
   3.____ 3 days
   4.____ 4 days
   5.____ 5 days or more

If your child HAS NOT had ANY belly aches or pain in the areas around or below the belly button in the past month (answered "never" to question 1 above), please go to Section C.

2. Does your child feel the belly ache, stomach ache or pain around or below his/her belly button <u>only</u>:

   a. When eating or right after eating

   0._____ No 1.___Yes

   b. (For girls only): When your child has her period

   0._____ No 1.____ Yes 2.___ My child has not started menstruation

3. How long has your child had a belly ache, stomach ache or pain around his/her belly button?

   1.____Less than a month
   2.____2 months
   3.____3 months
   4. ___4-11 months
   5. ___1 year or longer

Circle a number for your answer to each question below.					
**In the past month, when your child had a belly/stomach ache or pain around or below the belly button, how often:**	Never	Once in a while	Some-times	Most of the time	Always
4. Did the belly/stomach ache or pain happen around the time your child pooped?	0	1	2	3	4
5. Were your child's poops softer and more mushy or watery than usual?	0	1	2	3	4
6. Were your child's poops harder or lumpier than usual?	0	1	2	3	4
7. Did your child poop more often than usual?	0	1	2	3	4
8. Did your child poop less often than usual?	0	1	2	3	4

9. Does your child currently take medications for constipation/hard stools?
   1. ___ No
   2. ___ Yes
   3. ___ Don't know

   9a. If you answered Yes, how often does the belly ache, stomach ache or pain get better if your child uses these medications?
   a.____Never
   b.____Once in a while
   c.____Sometimes
   d.____Most of the time
   e.____Always

10. Does your child ever get an episode of **severe intense pain** around the belly button that hurts so much it makes your child **stop everything** that he or she is doing?
   0._____ No (*if no, please go to section C*)
   1._____ Yes

   10a. How long do these episodes of severe intense pain usually last?
   1. _____ Less than an hour
   2. _____ 1-2 hours
   3. _____ 3-4 hours
   4. _____ Most of the day
   5. _____ The whole day

   10b. IN THE PAST 6 MONTHS How often did your child get these episodes of severe intense pain?
   1._____1 time
   2._____2 times
   3._____3-5 times
   4._____6 or more times

10c. During the episode of severe intense pain, did your child have any of the following?

1. No appetite	0.______ No	1.______ Yes
2. Feeling sick to his/her stomach/nausea	0.______ No	1.______ Yes
3. Vomiting (throwing up)	0.______ No	1.______ Yes
4. Pale skin	0.______ No	1.______ Yes
5. Headache	0.______ No	1.______ Yes
6. Eyes sensitive to light	0.______ No	1.______ Yes

10d. When the pain stops, how long does it usually take for another episode of severe intense pain to start?

0.____ several minutes
1.____ several hours
2.____ several days
3.____ several weeks
4.____ several months

Section C. Bowel Movements ("Poop," "Stool," "Number 2")

This section asks about your child's bowel movements. There are many words for bowel movements, such as "poop," "stool," "BM's," and "going to the bathroom for number 2." Your family may use another special word when you talk about poops.

1. In the last month, how often did your child usually poop?
   1._____ 2 times a week or less often
   2._____ 3-6 times a week
   3._____ Once a day
   4._____ 2-3 times a day
   5._____ More than 3 times a day

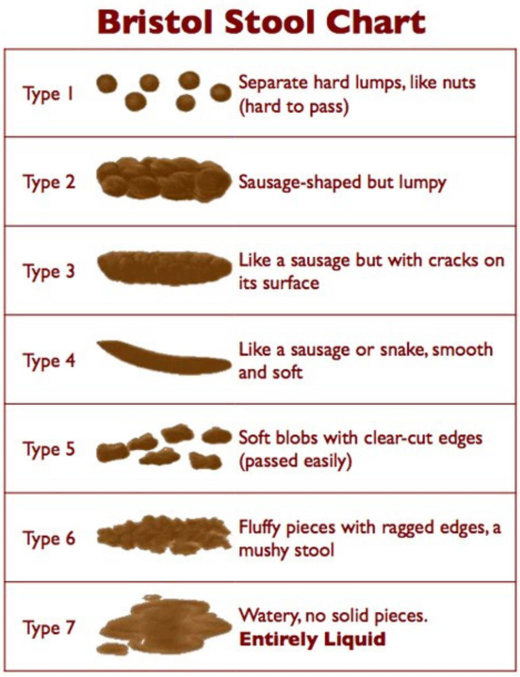

2. Look at the Bristol Stool Chart above. In the last month, what was your child's poop usually like?
   1. _______Type 1
   2. _______Type 2
   3. _______Type 3
   4. _______Type 4
   5. _______Type 5
   6. _______Type 6
   7. _______Type 7
   8. ________It depends (my child's poops are not always the same)

2a. If your child's poops were usually hard (type 1 or 2 on the scale above), for how long have they been hard?
   0.____Less than 1 month
   1.____1 month
   2.____2 or more months
   3.____My child's poops are not usually hard

3. In the last month, did it hurt when your child had a poop?
   0.____ No
   1.____ Yes

4. In the last month, did your child have a poop that was so big that it clogged the toilet?
   0.____ No
   1.____ Yes

5. Some children hold in their poop even when there is a toilet they could use. They may do this by stiffening their bodies or crossing their legs. In the last month, while at home, how often did your child try to hold in a poop?
   0.____ Never
   1.____ 1-3 times a month
   2.____ Once a week
   3.____ Several times a week
   4.____ Every day

6. Did a doctor or nurse ever examine your child and say that he or she had a huge poop inside?
   0.____ No
   1.____ Yes

7. In the last month, how often did your child poop in his or her pants?
   0.____ Never. *If never, please go to Section D.*
   1.____ Less than once a month
   2.____ 1-3 times a month
   3.____ Once a week
   4.____ Several times a week
   5.____Every day

   7a. When your child pooped in his or her pants, how much did it stain or soil their underwear?
      1.______ Underwear was only stained (no poop)
      2.______ Small amount of poop in underwear (less than a whole poop)
      3.______ Large amount of poop in underwear (a whole poop)

   7b. For how long has your child pooped in his or her pants?
      1.____ Less than a month
      2.____ 1 month
      3.____ 2 months
      4.____ 3-11 months
      5.____ 1 year or longer

Section D. Nausea and Vomiting

This section asks about feeling nauseated or vomiting. Nausea is when your child feels sick to their stomach or feels like they may throw up. There are many words for nausea, such as "upset stomach," "feeling queasy," or "feeling sick to the stomach."

1. In the past month, how often did your child feel nauseated or sick to the stomach?
   0.____ Never *(If never go to question 5)*
   1.____ 1-3 times a month
   2.____ Once a week
   3.____ Twice a week
   4.____ Three times a week or more often
   5.____ Every day

2. How long has your child felt nauseated or sick to the stomach?
   1.___Less than a month
   2.___2 months
   3.___3 months
   4. ___4-11 months
   5. ___1 year or longer

3. Does the nausea usually start after your child eats? 0.______ No 1.______ Yes

4. During the episode of nausea, did your child have any of the following?
   1. Vomiting 0.______No 1.______Yes
   2. Pain 0.______No 1.______Yes

   4 a. If your child has both pain and nausea, which one bothers your child the most? *Skip this question if your child does not have both pain and nausea.*
   1.______ Nausea
   2.______ Pain

The next questions are about vomiting. Vomiting is when food comes back up from your child's stomach out of your child's mouth. There are many words for vomiting such as "retching," "throwing up," "heaving" and "gagging." Your family may use another special word when you talk about vomiting.

5. In the past month, how often did your child vomit or throw up?
   0.____ Never *(If 'never' go to question 7)*
   1.____ 1-3 times a month
   2.____ Once a week
   3.____ Several times a week
   4.____ Every day

6. How long has your child had episodes of vomiting or throwing up?
   1.____ Less than a month
   2.____ 2 months
   3.____ 3 months
   4. ___ 4-11 months
   5. ___ 1 year or longer

7. IN THE PAST 6 MONTHS, how many times did your child vomit (throw up) *again and again without stopping for two hours or longer***?**

0.____ Never *(If never, please go to question 8)*
1.____ Once
2.____ 2 times
3.____ 3 times
4.____ 4 or more times

7a. For how long has your child had episodes of vomiting again and again without stopping?

1._____1 month or less
2._____ 2-3 months
3._____ 4-5 months
4._____ 6 months or longer

7b. Does your child usually feel nauseated or sick to his or her stomach when vomiting again and again without stopping?

0._____ No
1._____ Yes

7c. When the vomiting or throwing up stops, how long does it usually take for it to start again?

0._____ several minutes
1._____ several days
2._____ several weeks
3._____ several months or longer

8. In the past month, how often did food come back up into your child's mouth after eating?

0.____ Never *(If never, go to Question 9)*
1.____ 1-3 times a month
2.____ Once a week
3.____ Several times a week
4.____ Every day

8a. If food comes back into your child's mouth after eating, does this usually happen less than an hour after your child eats?

0._____ No
1._____ Yes

8b. Does it happen while your child is asleep?

0._____ No
1._____ Yes

8c. Does your child usually feel nauseated, sick to the stomach or vomit and throw up when this happens?

0._____ No
1._____ Yes

8d. What does your child usually do with the food that comes back up into his or her mouth?
0._____ Swallow it
1._____ Spit it out

9. Does your child ever make him or herself vomit or throw up (e.g., by sticking a finger in his or her throat)?
0._____ Never
1._____ Once in a while
2._____ Sometimes
3._____ Most of the time
4._____ Always

Section E. Other Symptoms

Circle a number for your child's answer to each question					
**In the past month, how often did your child:**	Never	1-3 times a month	Once a week	Several times a week	Every day
1. Burp (belch) *again and again* without wanting to?	0	1	2	3	4
2. Pass a lot of gas (fart) *very frequently*?	0	1	2	3	4
3. Develop a swollen belly during the day (a belly that definitely sticks out more than usual)?	0	1	2	3	4
4. Swallow or gulp extra air? (you might hear a clicking noise when your child swallows)	0	1	2	3	4
