# Supplementary material for: The Gut Microbiome Alterations in Pediatric Patients with Functional Abdominal Pain Disorders
Source: Microorganisms. 2021 Nov 15;9(11):2354. doi: 10.3390/microorganisms9112354 (PMC8622193; doi:10.3390/microorganisms9112354)
Supplement: Supplementary file 1 [file microorganisms-09-02354-s001.zip › Supplementary File S3.pdf]

Subject ID:______________________ Date:____ /____ /______

Please rate each gut problem on a scale from 0 to 10 where **0 = Does not bother me at all** and **10 = the most bothersome problem you can imagine**. Circle your response.

1) Difficulty passing stool

(Does not bother me) 0 1 2 3 4 5 6 7 8 9 10 (Most bothersome you can imagine)

2) Hard stools

(Does not bother me) 0 1 2 3 4 5 6 7 8 9 10 (Most bothersome you can imagine)

3) Loose stools

(Does not bother me) 0 1 2 3 4 5 6 7 8 9 10 (Most bothersome you can imagine)

4) Abdominal pain

(Does not bother me) 0 1 2 3 4 5 6 7 8 9 10 (Most bothersome you can imagine)

5) Vomiting

(Does not bother me) 0 1 2 3 4 5 6 7 8 9 10 (Most bothersome you can imagine)

6) Nausea

(Does not bother me) 0 1 2 3 4 5 6 7 8 9 10 (Most bothersome you can imagine)

7) Feeling full minutes after you start eating

(Does not bother me) 0 1 2 3 4 5 6 7 8 9 10 (Most bothersome you can imagine)

8) Not being able to finish your meal

(Does not bother me) 0 1 2 3 4 5 6 7 8 9 10 (Most bothersome you can imagine)

The answers above are for study-related purposes only and do not substitute for communication with your primary care physician (PCP). If your child scores greater than a score of 5 for any of the above questions, please discuss with your PCP. No outreach will be provided by the PCP or GI team in response to a positive symptom screen.
