# Supplementary material for: The Gut Microbiome Alterations in Pediatric Patients with Functional Abdominal Pain Disorders
Source: Microorganisms. 2021 Nov 15;9(11):2354. doi: 10.3390/microorganisms9112354 (PMC8622193; doi:10.3390/microorganisms9112354)
Supplement: Supplementary file 1 [file microorganisms-09-02354-s001.zip › Supplementary File S4.pdf]

SCORING INSTRUCTIONS FOR PARENT-REPORT FORM AND CHILD/ADOLESCENT SELF-REPORT FORM
For the Rome IV Diagnostic Questionnaire on Pediatric Gastrointestinal Symptoms for Children and Adolescents

I. Functional Dyspepsia

Functional Dyspepsia is diagnosed if child qualifies for post-prandial distress syndrome or epigastric pain syndrome or both.

<u>Post-prandial Distress Syndrome</u>

[(A 3) fullness "4 days a month" or more often, OR
(A4) satiation "4 days a month" or more often], AND
(A 7) Duration of upper abdominal pain or discomfort is "2 months" or longer.

<u>Epigastric Pain Syndrome</u>

[(A1) upper abdominal pain "4 days a month" or more often" OR
(A2) heartburn "4 days a month" or more often"], AND
(A 7) Duration of upper abdominal pain or discomfort is "2 months" or longer, AND
(A 8) Not related to a bowel movement "never" or "once in a while", AND
(A9-12) Not associated with change in stool form or frequency: "never" or "once in a while".

II. Irritable Bowel Syndrome

(B 1 or A1) abdominal pain "4 days a month" or more often AND
(B 3 or A7) abdominal pain is "2 months" or longer, AND
(B2a) Not exclusively associated with eating ('No'), AND
(B2b) for girls, not exclusively associated with menses ('No' or 'Not applicable'), AND
[At least one (A8-A12) OR (B4-B8) bowel symptoms "sometimes" or "more often", AND
(B9) for those who use laxatives (B 9 is "yes"), question (B9a) elimination of symptoms with laxatives must be answered 'never', 'once in a while', or 'sometimes' (i.e., NOT answered 'most of the time' or "Always').

III. Abdominal Migraine

(B10) Severe pain causing restriction in daily activities ("yes"), AND
(B10a) Pain lasts 1 hour or more, AND
(B10b) In the past 6 months, 2 or more episodes of severe pain, AND
[(B10c) Two or more of the following during pain episodes:
a. No appetite, OR
d. Pale skin, OR
b. Nausea, OR
e. Headache, OR
c. Vomiting, OR
f. Eyes sensitive to light], AND
(B10d) Pain episodes are separated by several weeks or longer.

IV Functional Abdominal Pain-nos
Lower abdominal location
(B1 OR A1) abdominal pain "4 days a month" or more often, AND
(B 3 or A7) abdominal pain is "2 months" or longer, AND
(B2a) pain is not exclusively associated with eating ('No'), AND
(B2b) in girls, pains is not exclusively associated with menses ('No' or 'Not applicable'), AND
Does not meet criteria for other functional gastrointestinal disorders associated with abdominal pain (e.g., functional dyspepsia, IBS, abdominal migraine).

V. Functional Constipation
Two or more of the following:
(C 1) Two or fewer stools per week, OR
(C3) painful stool] OR
(C4) Passage of very large stools, OR
(C5) Stool retention "once a week" or more often, OR
(C6) History of large fecal mass in rectum, OR
(C7) Soiling "once a week" or more often.
If child meets criteria for IBS, (B9) pain should improve with laxative use (B9).

VI. Non-retentive Fecal Incontinence
(C7) Soiling "once a week" or more often, AND
(C7a) Amount of stool is small or large (not just a stain), AND
(C7b) Soiling for 1 months or longer, AND
(C5) No evidence of fecal retention (C5 = never), AND
Does not meet criteria for functional constipation.

VII Functional Nausea
(D1) Nausea "twice a week" or more in the past 2 months, AND
(D2) Nausea for 2 months or longer, AND
(D3) Nausea not usually related to meal ("No"), AND
(D4) No vomiting during nausea episode ("No"), AND
(D4a) if co-morbid pain is present during nausea episode, nausea is more bothersome than pain.

VIII Functional Vomiting
(D5) Vomiting on average one or more times per week, AND
(D6) Vomiting for 2 months or longer, AND
(D7) Vomiting is not self-induced ("Never", "Once in a while"), AND
Child does not meet criteria for rumination.

IX. Cyclic Vomiting Syndrome
(D8) Two or more episodes of repeated vomiting in the past 6 months, AND
(D8b) Presence of nausea is "yes", AND
(D8c) vomit free intervals is "several weeks" or longer.

X. Adolescent Rumination Syndrome
(D9) Food comes back up "several times a week" or "every day", AND

(D9a) Episodes occur shortly after eating ("Yes"), AND
(D9b) Episodes do not occur during sleep ("No"), AND
(D9c) Episodes are not accompanied by nausea or vomiting ("No").

XI. Aerophagia

[(E1) belching "several times a week" or "every day", OR
(E2) flatus "several times a week" or "every day"] AND
(E3) Abdominal distention "several times a week" or "every day", AND
(E4) Swallowing air "several times a week" or "every day".
