# Supplementary material for: The Gut Microbiome Alterations in Pediatric Patients with Functional Abdominal Pain Disorders
Source: Microorganisms. 2021 Nov 15;9(11):2354. doi: 10.3390/microorganisms9112354 (PMC8622193; doi:10.3390/microorganisms9112354)
Supplement: Supplementary file 1 [file microorganisms-09-02354-s001.zip › Supplementary File S5.pdf]

sample-id	input	filtered	percentage of input passed filter	denoised	merged	percentage of input merged	non-chimeric	percentage of input non-chimeric
#q2:types	numeric	numeric	numeric	numeric	numeric	numeric	numeric	numeric
1-001	56654	20259	35.76	19577	18721	33.04	7790	13.75
1-002	237006	77136	32.55	74472	70499	29.75	27487	11.6
1-003	362368	124851	34.45	121868	116120	32.04	40983	11.31
1-004	86405	27120	31.39	25672	23544	27.25	8450	9.78
1-005	157070	54034	34.4	51815	48691	31	18904	12.04
1-006	277696	89227	32.13	86157	81325	29.29	29098	10.48
1-007	282485	98604	34.91	95855	91436	32.37	32260	11.42
1-008	123189	36343	29.5	34485	31920	25.91	14447	11.73
1-009	310579	105803	34.07	101778	94745	30.51	35219	11.34
1-011	209034	65459	31.32	63439	58848	28.15	17981	8.6
1-012	193143	63321	32.78	60435	56799	29.41	22100	11.44
1-015	116137	34843	30	33732	31537	27.15	11794	10.16
1-016	151068	52377	34.67	50689	47949	31.74	19317	12.79
1-017	168275	56512	33.58	54239	50968	30.29	22164	13.17
1-021	105499	35335	33.49	33486	30783	29.18	12216	11.58
1-024	74587	21967	29.45	20579	19104	25.61	10401	13.94
1-025	140254	46400	33.08	44279	41732	29.75	18639	13.29
1-027	84244	22040	26.16	21075	19597	23.26	8012	9.51
1-029	320853	109268	34.06	105653	99754	31.09	36534	11.39
1-030	56971	17761	31.18	16977	15551	27.3	7625	13.38
1-033	160488	55038	34.29	52821	49466	30.82	19626	12.23
1-034	311356	105406	33.85	102520	95793	30.77	34419	11.05
1-036	118907	37687	31.69	36090	33434	28.12	14975	12.59
1-039	256954	80070	31.16	77046	71371	27.78	27105	10.55
1-043	40595	12729	31.36	11892	10879	26.8	6190	15.25
1-044	231271	77604	33.56	74608	69812	30.19	20776	8.98
1-046	181085	64548	35.65	62586	58301	32.2	22092	12.2
1-055	66354	17919	27.01	16910	15706	23.67	8752	13.19
2-001a	188040	60853	32.36	58482	55039	29.27	21641	11.51

2-001b	218653	71216	32.57	68747	63831	29.19	20463	9.36
3-001	457973	147707	32.25	142848	132917	29.02	57647	12.59
3-002	170829	57807	33.84	55430	51419	30.1	19042	11.15
3-003	96031	30545	31.81	29051	27057	28.18	12325	12.83
3-004	119690	38876	32.48	36903	34337	28.69	14631	12.22
3-007	157625	51301	32.55	48745	44796	28.42	16403	10.41
3-008	168680	50049	29.67	47661	44135	26.16	18786	11.14
3-013	123796	42099	34.01	40234	37389	30.2	14980	12.1
3-014	302121	102870	34.05	99553	94389	31.24	32575	10.78
3-015	366016	122190	33.38	118446	112281	30.68	37240	10.17
3-016	251335	85205	33.9	82131	76515	30.44	28367	11.29
3-017	85773	26199	30.54	24703	22548	26.29	11577	13.5
3-019	192372	60894	31.65	58160	53909	28.02	20657	10.74
4-001	649487	201050	30.96	196576	188015	28.95	58101	8.95
4-002	73660	19979	27.12	19090	17294	23.48	8575	11.64
4-003	140668	46637	33.15	44750	41588	29.56	14488	10.3
4-004	99631	30393	30.51	29349	27008	27.11	9064	9.1
4-005	276802	79062	28.56	75960	70432	25.44	22501	8.13
4-006	235197	71294	30.31	68302	63591	27.04	24745	10.52
4-007	105816	33444	31.61	31799	29508	27.89	12134	11.47
4-008	255808	76544	29.92	73433	68078	26.61	27032	10.57
4-009	164004	52932	32.27	50329	46837	28.56	18180	11.09
4-010	158608	45310	28.57	42708	39889	25.15	17090	10.77
4-011	195075	65643	33.65	62888	59240	30.37	21179	10.86
4-012	120030	38322	31.93	36607	33677	28.06	15005	12.5
5-001	319920	103648	32.4	98352	91058	28.46	36412	11.38
5-002	365294	118117	32.33	113051	105020	28.75	38278	10.48
5-003	36224	11215	30.96	10583	9521	26.28	5273	14.56
5-004	92849	30729	33.1	29329	27209	29.3	11945	12.86
5-005	138875	44402	31.97	42683	39793	28.65	17426	12.55
5-011	67535	19185	28.41	18084	16533	24.48	8608	12.75
5-012	94605	30598	32.34	29521	28041	29.64	10617	11.22
5-013	63411	22261	35.11	21604	20359	32.11	7983	12.59
5-014	130531	39887	30.56	38177	36048	27.62	16054	12.3

5-015	82999	23882	28.77	22431	20952	25.24	11229	13.53
5-016	149582	46166	30.86	43484	39614	26.48	18744	12.53
5-017	202351	64795	32.02	61722	57664	28.5	24688	12.2
5-018	69160	22390	32.37	21104	19053	27.55	9038	13.07
5-020	47662	12591	26.42	11775	10681	22.41	5751	12.07
5-021	49795	16733	33.6	15813	14609	29.34	6380	12.81
5-022	124284	36285	29.2	34365	31853	25.63	14213	11.44
5-023	152212	50835	33.4	48898	45999	30.22	17321	11.38
5-024	87347	28954	33.15	27725	25409	29.09	11199	12.82
5-025	308132	106851	34.68	102440	94458	30.66	32250	10.47
5-026	231801	76398	32.96	72742	66327	28.61	24641	10.63
6-001	522167	170746	32.7	165043	154179	29.53	47416	9.08
6-002	205461	54388	26.47	51673	47509	23.12	19989	9.73
6-003	126212	39725	31.47	37162	34129	27.04	14435	11.44
6-009	184028	54586	29.66	51610	46872	25.47	18392	9.99
6-015	221624	74909	33.8	71391	65563	29.58	24915	11.24
6-019	197157	60640	30.76	57411	53089	26.93	23044	11.69
6-021	67728	21937	32.39	20787	19219	28.38	8553	12.63
6-022	59426	18878	31.77	17626	16194	27.25	7619	12.82
6-023	53464	16109	30.13	15045	13510	25.27	6995	13.08
1-001c	152834	115470	75.55	114592	113284	74.12	38000	24.86
1-002c	117329	80834	68.9	78697	75465	64.32	17658	15.05
1-003c	129506	95285	73.58	93391	89735	69.29	25341	19.57
1-005c	100988	72125	71.42	71067	67580	66.92	17509	17.34
1-006c	117049	85874	73.37	83992	81158	69.34	19813	16.93
1-007c	107990	80832	74.85	79415	77014	71.32	26120	24.19
1-011c	135983	99407	73.1	97820	94232	69.3	22531	16.57
1-015c	120457	90236	74.91	88589	84877	70.46	18319	15.21
1-016c	82418	61900	75.1	60764	58265	70.69	15479	18.78
1-017c	103143	72467	70.26	70672	66893	64.85	17919	17.37
1-021c	94852	66516	70.13	64696	61739	65.09	15076	15.89
1-024c	60140	38373	63.81	37004	35171	58.48	10372	17.25
1-025c	84014	63856	76.01	62565	60195	71.65	17412	20.73
1-029c	86866	64192	73.9	63565	62456	71.9	28058	32.3

1-030c	89333	67257	75.29	65974	63076	70.61	16712	18.71
1-033c	96314	72465	75.24	71358	69522	72.18	27759	28.82
1-039c	125168	90735	72.49	88908	84682	67.65	19684	15.73
1-043c	108938	77243	70.91	74112	69760	64.04	18769	17.23
1-044c	121495	90771	74.71	89215	84406	69.47	19467	16.02
1-046c	84023	58205	69.27	57290	55319	65.84	12606	15
3-001c	108017	81587	75.53	79779	74540	69.01	17452	16.16
3-002c	118982	86687	72.86	84409	79999	67.24	19037	16
3-003c	122075	89473	73.29	87076	83320	68.25	22144	18.14
3-007c	112646	81555	72.4	79650	75253	66.8	17655	15.67
3-008c	149279	111264	74.53	109025	103428	69.29	26128	17.5
3-014c	113679	84066	73.95	81910	77669	68.32	20090	17.67
3-015c	116588	84160	72.19	81735	78137	67.02	19865	17.04
3-016c	102771	73021	71.05	71209	67445	65.63	20565	20.01
3-019c	141259	102364	72.47	98262	90528	64.09	21481	15.21
4-001c	128063	96249	75.16	94967	91985	71.83	21489	16.78
4-002c	146598	109616	74.77	106535	100204	68.35	24104	16.44
4-003c	139281	100600	72.23	97710	92667	66.53	23363	16.77
4-004c	118382	87262	73.71	85418	80459	67.97	16149	13.64
4-005c	120912	84465	69.86	81480	76627	63.37	15841	13.1
4-006c	110687	80471	72.7	78534	74645	67.44	22300	20.15
4-007c	80878	55977	69.21	54791	52319	64.69	12723	15.73
4-008c	102534	76718	74.82	75365	72536	70.74	19482	19
4-009c	116559	86593	74.29	84657	81035	69.52	21413	18.37
4-010c	110103	80968	73.54	78444	74901	68.03	21899	19.89
4-011c	111185	80742	72.62	78760	75122	67.56	19429	17.47
4-012c	126880	91765	72.32	89926	86017	67.79	21774	17.16
5-001c	118938	87806	73.83	85714	81647	68.65	19858	16.7
5-002c	122491	84691	69.14	82543	79347	64.78	20586	16.81
5-003c	96277	67252	69.85	65891	62954	65.39	16591	17.23
5-004c	121552	89486	73.62	86832	82312	67.72	20992	17.27
5-005c	127514	94405	74.04	92570	87996	69.01	20637	16.18
5-011c	129495	95589	73.82	92333	87368	67.47	25104	19.39
5-012c	125223	91282	72.9	89607	85909	68.6	23096	18.44

5-013c	136698	100574	73.57	98837	95266	69.69	27991	20.48
5-014c	101158	72739	71.91	71297	68055	67.28	15800	15.62
5-015c	112049	82496	73.62	79740	75230	67.14	18008	16.07
5-016c	92451	65835	71.21	63934	59742	64.62	13768	14.89
5-017c	108687	80356	73.93	78203	74616	68.65	20861	19.19
5-018c	118553	88726	74.84	86748	83428	70.37	22477	18.96
5-022c	106555	77916	73.12	75715	72521	68.06	17886	16.79
5-023c	114818	84042	73.2	82672	80236	69.88	15130	13.18
5-024c	135000	101558	75.23	99263	93619	69.35	22655	16.78
5-025c	130111	97710	75.1	94926	88177	67.77	17672	13.58
5-026c	118458	87370	73.76	85013	80055	67.58	21197	17.89
6-001c	101834	72468	71.16	70801	67098	65.89	14569	14.31
6-002c	108664	77469	71.29	75749	73073	67.25	22223	20.45
6-003c	123459	91714	74.29	90206	87545	70.91	19097	15.47
6-019c	89041	67757	76.1	66511	64571	72.52	18262	20.51
6-023c	97258	64634	66.46	63050	60200	61.9	13637	14.02
1-001d	134429	98645	73.38	97304	94204	70.08	23555	17.52
1-002d	126790	89847	70.86	87577	82639	65.18	16491	13.01
1-003d	164231	112122	68.27	109751	103444	62.99	23963	14.59
1-005d	127531	92673	72.67	90878	84695	66.41	17094	13.4
1-006d	146012	103333	70.77	100341	94114	64.46	23056	15.79
1-007d	152434	106358	69.77	104266	98522	64.63	20655	13.55
1-008d	135942	92703	68.19	90260	85300	62.75	18733	13.78
1-011d	121678	86551	71.13	84267	78953	64.89	13889	11.41
1-015d	157821	114520	72.56	112581	107313	68	20927	13.26
1-016d	139687	96215	68.88	94176	89322	63.94	21387	15.31
1-017d	113880	80101	70.34	77277	72592	63.74	18684	16.41
1-021d	117627	82513	70.15	79440	74634	63.45	16069	13.66
1-024d	116113	77744	66.96	74782	70058	60.34	17336	14.93
1-025d	124674	92452	74.15	90100	84964	68.15	19491	15.63
1-029d	152405	112600	73.88	110055	103177	67.7	21364	14.02
1-030d	112944	83986	74.36	82460	78335	69.36	20315	17.99
1-033d	120592	89668	74.36	87852	83859	69.54	24478	20.3
1-036c	158489	117620	74.21	115463	109196	68.9	21980	13.87

1-039d	124496	87123	69.98	85166	80017	64.27	15256	12.25
1-043d	112182	79026	70.44	76865	72827	64.92	13978	12.46
1-044d	106961	78297	73.2	76603	72199	67.5	16525	15.45
1-046d	128996	93599	72.56	91704	86335	66.93	17679	13.71
1-055c	104250	71835	68.91	69543	64947	62.3	14927	14.32
2-001d	160056	112758	70.45	110287	104832	65.5	19075	11.92
3-001d	104462	78358	75.01	76310	70897	67.87	15976	15.29
3-002d	117707	86979	73.89	84880	79081	67.18	16710	14.2
3-003d	119895	87408	72.9	85079	80042	66.76	19416	16.19
3-004d	120132	87136	72.53	85054	80345	66.88	18167	15.12
3-007d	138232	98346	71.15	95450	88826	64.26	18145	13.13
3-008d	137325	102352	74.53	99977	94825	69.05	23546	17.15
3-013c	124209	89725	72.24	86913	81463	65.59	20039	16.13
3-013d	135478	100152	73.92	97816	92737	68.45	23576	17.4
3-014d	126257	89349	70.77	87165	82624	65.44	21332	16.9
3-015d	138378	98767	71.37	95814	91359	66.02	19003	13.73
3-016d	134432	98072	72.95	95568	90510	67.33	21082	15.68
3-019d	174534	124620	71.4	120536	111636	63.96	24725	14.17
4-001d	117678	84894	72.14	83347	79886	67.89	16325	13.87
4-002d	96828	70725	73.04	68375	63610	65.69	15081	15.58
4-003d	80537	55442	68.84	52789	49134	61.01	11835	14.7
4-004d	101360	73004	72.02	70618	66569	65.68	15952	15.74
4-005d	90439	65655	72.6	64232	60177	66.54	13385	14.8
4-006d	134654	94383	70.09	91416	85440	63.45	20729	15.39
4-007d	109822	78117	71.13	76015	71009	64.66	14323	13.04
4-008d	122370	87881	71.82	85703	81038	66.22	19188	15.68
4-009d	109692	72949	66.5	70780	66981	61.06	17213	15.69
4-010d	116019	82448	71.06	79425	74807	64.48	17039	14.69
4-011d	99776	69369	69.52	67152	62973	63.11	14869	14.9
4-012d	158141	112460	71.11	110310	105678	66.83	24167	15.28
5-001d	177445	130842	73.74	127322	119136	67.14	25343	14.28
5-002d	133958	98347	73.42	95362	88570	66.12	19332	14.43
5-003d	114242	83493	73.08	81272	76407	66.88	18329	16.04
5-004d	131220	96031	73.18	92450	85462	65.13	21581	16.45

5-011d	130943	94517	72.18	90519	84177	64.29	21289	16.26
5-012d	160374	115208	71.84	113262	108760	67.82	26086	16.27
5-013d	150302	111350	74.08	109284	103664	68.97	25549	17
5-016d	179436	130281	72.61	126883	117228	65.33	25970	14.47
5-017d	190797	133892	70.18	129252	120348	63.08	27782	14.56
5-018d	207274	152063	73.36	147923	138064	66.61	29488	14.23
5-022d	138537	97872	70.65	94051	87238	62.97	19996	14.43
5-023d	225270	163317	72.5	159938	152387	67.65	25241	11.2
5-024d	171566	128365	74.82	125060	116080	67.66	23214	13.53
5-025d	133197	97789	73.42	95240	89218	66.98	19607	14.72
6-001d	157687	113529	72	110417	102808	65.2	21184	13.43
6-002d	135284	93020	68.76	89916	85013	62.84	19633	14.51
6-003d	127049	90912	71.56	88690	84668	66.64	14982	11.79
6-019d	154946	109829	70.88	107115	101678	65.62	18437	11.9
