# Supplementary material for: The Gut Microbiome Alterations in Pediatric Patients with Functional Abdominal Pain Disorders
Source: Microorganisms. 2021 Nov 15;9(11):2354. doi: 10.3390/microorganisms9112354 (PMC8622193; doi:10.3390/microorganisms9112354)
Supplement: Supplementary file 1 [file microorganisms-09-02354-s001.zip › Supplementary File S6.pdf]

	**Symptomatic**		**Asymptomatic**		**Fisher's Exact Test (*p*-values)**	**Significance**
**Symptoms**	**FAPDs**	**Control**	**FAPDs**	**Control**		
Difficulty passing stools	25 (89.3)	24 (44.4)	3 (10.7)	30 (55.6)	**< 0.001**	**Yes**
Hard stools	21 (75.0)	33 (61.1)	7 (25.0)	21 (38.9)	**0.231**	**No**
Loose stools	18 (64.3)	27 (50.0)	10 (35.7)	27 (50.0)	**0.249**	**No**
Abdominal pain	27 (96.4)	17 (31.5)	1 (3.6)	37 (68.5)	**< 0.001**	**Yes**
Vomiting	11 (39.3)	8 (14.8)	17 (60.7)	46 (85.2)	**0.025**	**Yes**
Nausea	23 (82.1)	17 (31.5)	5 (17.9)	37 (68.5)	**< 0.001**	**Yes**
Feel full	18 (64.3)	20 (37.0)	10 (35.7)	34 (63)	**0.022**	**Yes**
Not finish	23 (82.1)	17 (31.5)	5 (17.9)	37 (68.5)	**< 0.001**	**Yes**
