# Supplementary material for: The Gut Microbiome Alterations in Pediatric Patients with Functional Abdominal Pain Disorders
Source: Microorganisms. 2021 Nov 15;9(11):2354. doi: 10.3390/microorganisms9112354 (PMC8622193; doi:10.3390/microorganisms9112354)
Supplement: Supplementary file 1 [file microorganisms-09-02354-s001.zip › Supplementary File S7.pdf]

Variable	Feature	Value	Coefficient	N	N.not.0	P.Value	Q.Value
Group	d__Bacteria;p__Verrucomicrobiota	FAPDs	-0.026738041	213	125	0.000764991	0.005354939
Group	d__Bacteria;p__Firmicutes	FAPDs	-0.035286785	213	213	0.009344018	0.03448996
Group	d__Bacteria;p__Bacteroidota	FAPDs	0.036615934	213	213	0.009854274	0.03448996
Group	d__Bacteria;p__Patescibacteria	FAPDs	-0.011339167	213	98	0.013953774	0.043411742
School term	d__Bacteria;p__Patescibacteria	Vacation	0.019857981	213	98	2.34E-05	0.00037461
School term	d__Bacteria;p__Fusobacteriota	Vacation	0.023537683	213	92	2.68E-05	0.00037461
School term	Unassigned;__	Vacation	0.012526151	213	64	8.01E-05	0.000747297
School term	d__Bacteria;p__Spirochaetota	Vacation	0.003690758	213	33	0.001844561	0.010329544
School term	d__Bacteria;p__Proteobacteria	Vacation	0.026617022	213	212	0.006235589	0.029099415
