# Supplementary material for: The Gut Microbiome Alterations in Pediatric Patients with Functional Abdominal Pain Disorders
Source: Microorganisms. 2021 Nov 15;9(11):2354. doi: 10.3390/microorganisms9112354 (PMC8622193; doi:10.3390/microorganisms9112354)
Supplement: Supplementary file 1 [file microorganisms-09-02354-s001.zip › Supplementary File S8.pdf]

	Variable	Feature	Value	Coefficient	N	N.not.0	P.Value	Q.Value
1	Group	Lachnospiraceae_ND3007_group	FAPDs	-0.029495425	213	149	1.24E-07	2.86E-05
2	Group	Ruminococcus	FAPDs	-0.037927312	213	163	1.55E-06	0.0001734
3	Group	UCG-003	FAPDs	-0.028022565	213	122	1.88E-06	0.0001734
4	Group	Lachnospiraceae_NK4A136_group	FAPDs	-0.036178951	213	172	1.29E-05	0.0005435
5	Group	FCS020_group	FAPDs	-0.018135944	213	120	3.86E-05	0.0011149
6	Group	CAG-56	FAPDs	-0.018979783	213	71	5.35E-05	0.0014033
7	Group	Candidatus_Stoquefichus	FAPDs	0.014118554	213	36	5.47E-05	0.0014033
8	Group	Christensenellaceae_R-7_group	FAPDs	-0.026879815	213	177	6.15E-05	0.0014199
9	Group	Eubacterium	FAPDs	0.005688827	213	13	6.15E-05	0.0014199
10	Group	Bacteroides	FAPDs	0.119127237	213	212	7.05E-05	0.0015519
11	Group	Erysipelotrichaceae_UCG-003	FAPDs	-0.023549087	213	137	0.0001234	0.0024791
12	Group	;g__uncultured	FAPDs	-0.011540048	213	27	0.0003418	0.0047858
13	Group	g__Mitochondria	FAPDs	-0.001803269	213	6	0.0004311	0.0055327
14	Group	Sutterella	FAPDs	-0.026977059	213	79	0.0005201	0.0064943
15	Group	Coprococcus	FAPDs	-0.021395517	213	174	0.0009793	0.0110351
16	Group	Neisseria	FAPDs	-0.027535808	213	85	0.0015308	0.0167061
17	Group	Lachnospiraceae_UCG-010	FAPDs	-0.008055101	213	33	0.0017488	0.0181399
18	Group	Enterobacteriaceae;__	FAPDs	-0.027444005	213	152	0.0017669	0.0181399
19	Group	Akkermansia	FAPDs	-0.02567955	213	116	0.0018474	0.01816
20	Group	_UCG-009	FAPDs	-0.004941857	213	20	0.0020066	0.0186209
21	Group	Lachnospiraceae_UCG-001	FAPDs	-0.012861216	213	72	0.0020552	0.0186209
22	Group	Porphyromonas	FAPDs	-0.018937999	213	90	0.0020556	0.0186209
23	Group	Subdoligranulum	FAPDs	-0.023889331	213	194	0.0022979	0.0204162
24	Group	Dialister	FAPDs	-0.024308842	213	159	0.0023827	0.0207697
25	Group	UCG-002	FAPDs	-0.031163798	213	158	0.0024392	0.020869
26	Group	[Eubacterium]_xylanophilum_group	FAPDs	-0.014993882	213	104	0.0025435	0.0213652
27	Group	[Clostridium]_innocuum_group	FAPDs	0.012062481	213	102	0.0029145	0.0236228
28	Group	[Eubacterium]_ventriosum_group	FAPDs	-0.013281023	213	156	0.0032517	0.0259018
29	Group	Fusicatenibacter	FAPDs	-0.025193424	213	189	3.43E-03	2.69E-02
30	Group	Monoglobus	FAPDs	-0.01387037	213	167	3.87E-03	0.0298119
31	Group	Anaerofustis	FAPDs	0.002238282	213	6	4.57E-03	0.0346158
32	Group	Eisenbergiella	FAPDs	0.01111388	213	55	4.72E-03	0.0351824
33	Group	Faecalibacterium	FAPDs	-0.032405227	213	208	5.98E-03	0.0418385

34	Group	Acetanaerobacterium	FAPDs	0.002316701	213	19	6.37E-03	0.0428182
35	Group	Eggerthella	FAPDs	0.009001483	213	106	6.39E-03	0.0428182
36	Group	Solobacterium	FAPDs	-0.011008284	213	78	6.61E-03	0.0436107
37	Group	Romboutsia	FAPDs	-0.017168158	213	156	6.98E-03	0.0454005
38	Group	Veillonella	FAPDs	-0.033712912	213	124	7.35E-03	0.0471869
39	School term	Enterobacteriaceae;__	Vacation	0.048942263	213	152	5.78E-08	2.67E-05
40	School term	Neisseria	Vacation	0.042965906	213	85	1.25E-06	0.0001734
41	School term	Solobacterium	Vacation	0.019184034	213	78	3.54E-06	0.0002728
42	School term	Porphyromonas	Vacation	0.028087586	213	90	6.92E-06	0.000433
43	School term	Bacteroides	Vacation	-0.135430833	213	212	7.50E-06	0.000433
44	School term	Megasphaera	Vacation	0.027242411	213	95	1.11E-05	0.0005312
45	School term	Moraxella	Vacation	0.019881483	213	78	1.15E-05	0.0005312
46	School term	Alloprevotella	Vacation	0.023603048	213	83	1.70E-05	0.0006532
47	School term	Granulicatella	Vacation	0.026142153	213	86	2.88E-05	0.0010006
48	School term	Atopobium	Vacation	0.028952297	213	80	3.03E-05	0.0010006
49	School term	Parvimonas	Vacation	0.018011122	213	82	3.71E-05	0.0011149
50	School term	Veillonella	Vacation	0.050102609	213	124	8.50E-05	0.0017842
51	School term	Lactobacillus	Vacation	0.051356237	213	103	0.0001348	0.0025956
52	School term	Alloscardovia	Vacation	0.027406326	213	79	0.0001598	0.0029536
53	School term	Fusobacterium	Vacation	0.019537282	213	90	0.0001955	0.0033747
54	School term	Gemella	Vacation	0.020119509	213	84	0.0002037	0.0033747
55	School term	Stenotrophomonas	Vacation	0.020731485	213	80	0.0002045	0.0033747
56	School term	Saccharimonadaceae;g__TM7x	Vacation	0.014326151	213	94	0.0002207	0.0035165
57	School term	Peptoniphilus	Vacation	0.020246875	213	81	0.0002982	0.0045919
58	School term	g__Mitochondria	Vacation	0.001844637	213	6	0.0003356	0.0047858
59	School term	Actinomyces	Vacation	0.017262775	213	79	0.0003396	0.0047858
60	School term	Peptostreptococcus	Vacation	0.021625287	213	80	0.0003566	0.004846
61	School term	d__Bacteria;p__Firmicutes;c__Clostridia;o__Lachnospirales;f__Lachnospiraceae;g__Lachnoanaerobaculum	Vacation	0.003459835	213	12	0.0004254	0.0055327
62	School term	Klebsiella	Vacation	0.027506904	213	87	0.0005619	0.0068319
63	School term	Streptococcus	Vacation	0.035281599	213	199	0.0005996	0.0071029
64	School term	Bacilli;__;__;__	Vacation	0.013448316	213	69	0.0006708	0.0077478
65	School term	Prevotella	Vacation	0.099011566	213	130	0.0015549	0.0167061
66	School term	Treponema	Vacation	0.003690758	213	33	0.0018446	0.01816

67	School term	[Eubacterium]_nodatum_group	Vacation	0.009232286	213	74	0.0020101	0.0186209
68	School term	Absconditabacteriales_(SR1)	Vacation	0.003050804	213	29	0.002788	0.0230007
69	School term	Holdemania	Vacation	-0.008067554	213	126	0.0050945	0.03736
70	School term	Weissella	Vacation	0.002571863	213	4	0.0052146	0.037643
71	School term	Haemophilus	Vacation	0.019701054	213	123	0.0057193	0.0406507
72	School term	Selenomonas	Vacation	0.003123609	213	22	0.006348	0.0428182
