# Supplementary figures and images for: The Gut Microbiome Alterations in Pediatric Patients with Functional Abdominal Pain Disorders
Source: Microorganisms. 2021 Nov 15;9(11):2354. doi: 10.3390/microorganisms9112354 (PMC8622193; doi:10.3390/microorganisms9112354)

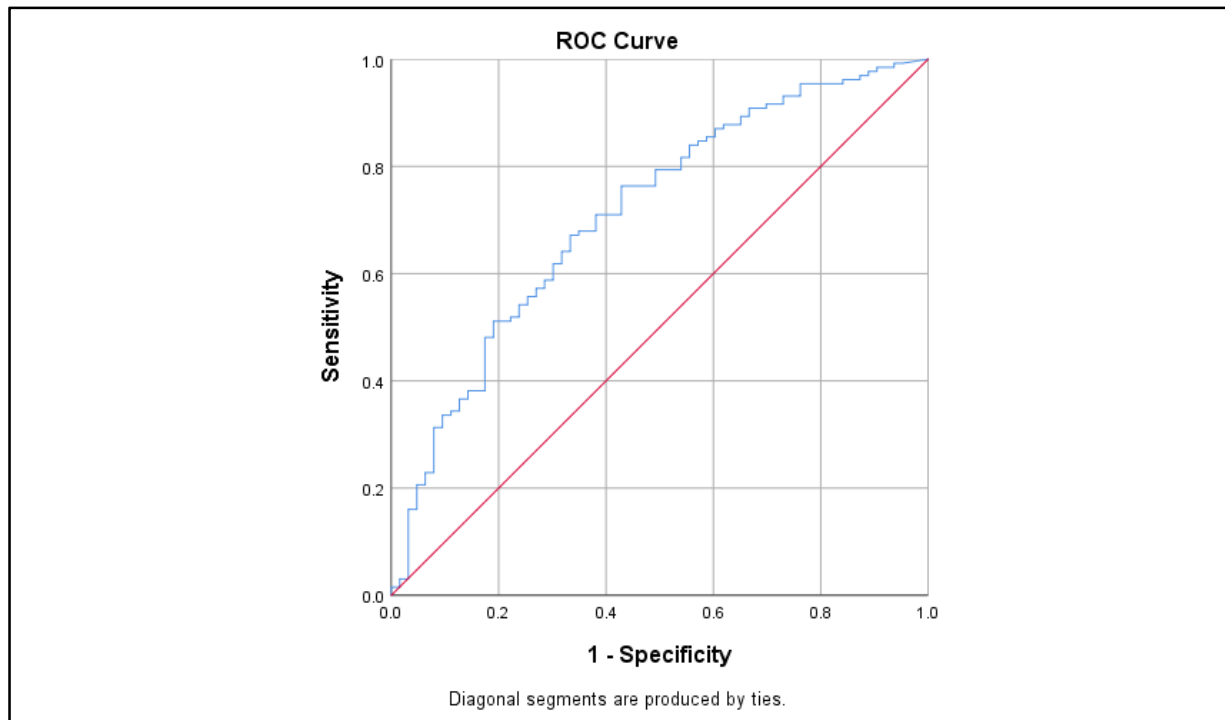

**(A)**

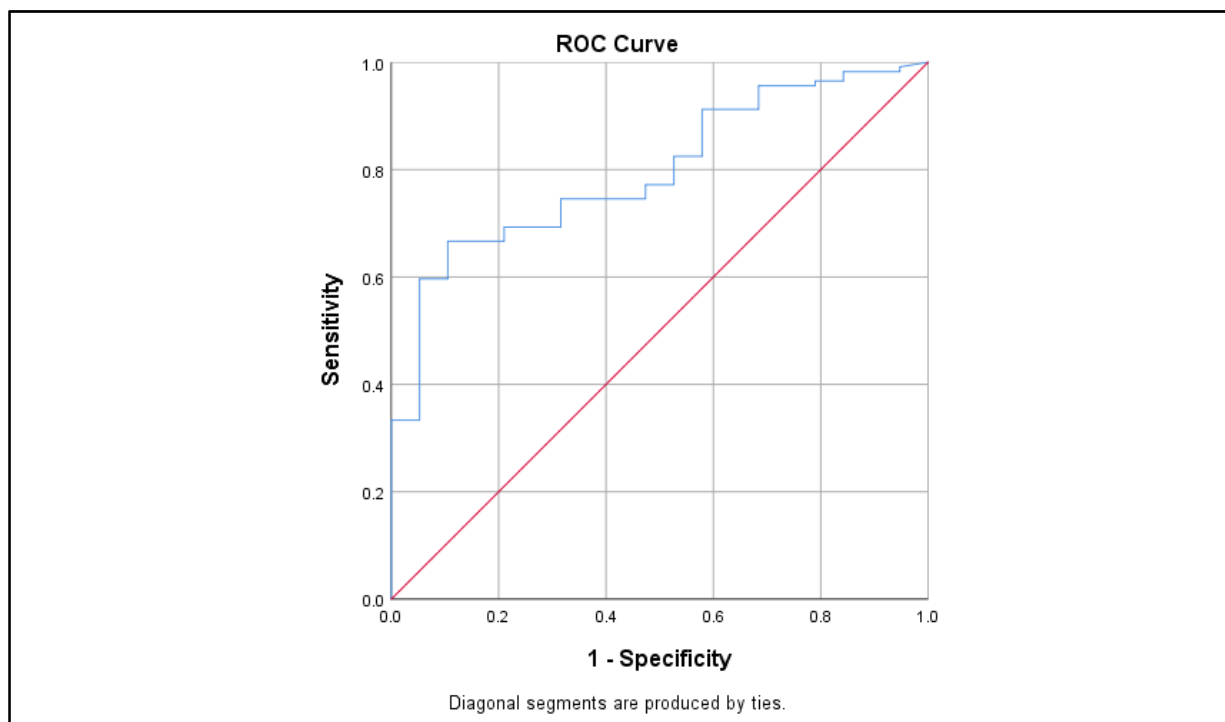

**(B)**

Supplement: Supplementary file 1 [file microorganisms-09-02354-s001.zip › Supplementary File S11.pdf]

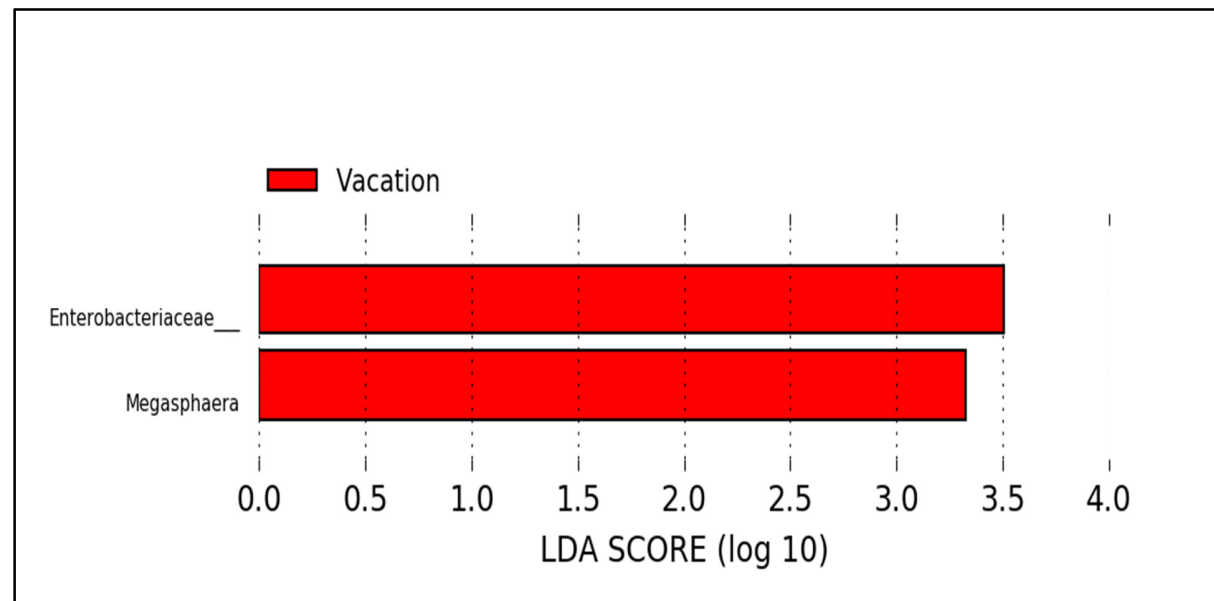

Supplement: Supplementary file 1 [file microorganisms-09-02354-s001.zip › Supplementary File S9.pdf]
